# Testing a Deliberative Democracy Engagement Intervention to Increase Guideline-Concordance Among Oral Health Providers: Results from the DISGO Cluster-Randomized, Stepped-Wedge Trial

**DOI:** 10.21203/rs.3.rs-2757518/v1

**Published:** 2023-05-04

**Authors:** Deborah Polk, Nilesh H. Shah, Tim Dawson, Inga Gruß, Daniel J. Pihlstrom, Charles D. Kaplan, Erick G. Guerrero, Jeffrey L. Fellows

**Affiliations:** University of Pittsburgh School of Dental Medicine; University of Pittsburgh School of Dental Medicine; The Art of Democracy; Kaiser Permanente Center for Health Research; Permanente Dental Associates; Sunrise Community Counseling Center; Research to End Healthcare Disparities Corp.; Kaiser Permanente Center for Health Research

**Keywords:** Implementation strategy, oral health, pit and fissure sealants, deliberative democracy, guideline adherence, provider autonomy

## Abstract

**Background:**

Dental caries affects billions worldwide and in the U.S. is among the most prevalent noncommunicable diseases in both children and adults. Early in the caries process, it can be arrested by dental sealants, which are non-invasive and thus tooth sparing, however, few dentists have adopted this approach. Deliberative engagement processes enable participants to engage with diverse perspectives on a policy issue and develop and share with policy makers informed opinions about the policy issue. We examined the effects of a deliberative engagement process on the ability of oral health providers to endorse implementation interventions and to apply dental sealants.

**Methods:**

Using a stepped wedge design, 16 dental clinics were cluster randomized, and 680 providers and staff were exposed to a deliberative engagement process that included an introductory session, workbook, facilitated small group deliberative forum, and post-forum survey. Forum participants were assigned to forums to ensure diverse role representation. Mechanisms of action examined included sharing voice and diversity of opinion. Three months after each clinic’s forum, the clinic manager was interviewed about implementation interventions deployed. There were 98 clinic-months in the non-intervention period and 101 clinic-months in the intervention period.

**Results:**

Compared with providers and staff in small clinics, providers and staff in medium and large clinics more strongly agreed that their clinic should adopt two of the three proposed implementation interventions targeting the first barrier and one of the two proposed implementation interventions targeting the second barrier. Compared with the non-intervention period, in the intervention period providers did not place more sealants on occlusal non-cavitated carious lesions. Survey respondents reported sharing both promotive and prohibitive voice. From the beginning to the end of the forums, most forum participants did not change their opinions about the possible implementation interventions. At the end of the forums, there was no significant within-group variability in implementation interventions endorsed.

**Conclusions:**

Deliberative engagement intervention may help clinic leadership identify implementation interventions when there is a challenging problem, a network of semi-autonomous clinics, and autonomous providers within those clinics. It remains to be determined whether there is a range of perspectives within clinics.

**Trial Registration:**

This project is registered at ClinicalTrials.gov with ID NCT04682730. The trial was first registered on 12/18/2020. https://clinicaltrials.gov/ct2/show/NCT04682730

## Background

Despite organizations’ adoption of guidelines, health care providers can have difficulty putting the guidelines into practice, creating an evidence-to-practice gap. Challenges can come from multiple sources including practice settings with networked clinics that are accustomed to practicing semi-autonomously and providers who are also accustomed to significant autonomy (1, 2, 3, 4). These two sources of autonomy may make it difficult to develop implementation strategies. In addition, increasing guideline adherence may require participation from people holding diverse roles within the clinic. However, adopting decision-making processes that capitalize on these features may transform barriers into facilitators.

An approach to identifying implementation interventions and increasing guideline adherence that may capitalize on semi-autonomous clinics, providers with autonomy, and staff with diverse roles is grounded in the work of Kurt Lewin. In the 1930s and 1940s, Lewin and colleagues demonstrated that when factory workers in an industrial setting used a democratic process to make decisions about their rate of work, their productivity increased (5, 6). Based on this evidence, we sought to determine whether engaging providers and staff in a democratic process to endorse implementation interventions to adopt could increase their guideline adherence.

To expand on the mechanisms of action occurring during a democratic process, we were guided by theories of deliberative democracy. Deliberative processes enable interested parties to develop informed opinions by giving them exposure to background information and the opportunity to engage with the diverse perspectives of other interested parties (7). Collective wisdom emerges through inclusive processes that involve individuals learning from one another. This collective wisdom, in turn, becomes a resource individuals and decision makers can draw on as they seek to form opinions about, decide on, and take actions to address problems (8).

The process of deliberation takes place in facilitated small group forums. This feature may be one reason why a deliberative process could work well in semi-autonomous clinics. Deliberative forums can be structured around clinics, enabling each clinic to identify an implementation strategy that works best for them. Thus, we hypothesized that deliberative processes may be well-suited to decision making in semi-autonomous clinics.

The deliberative process involves engaging the people who will be affected by the selection of an action. Including both clinic providers and staff may be particularly effective because they represent different roles in the workplace, each with their own perspective (9, 10, 11). In addition, sharing their informed opinions with clinic leaders gives them voice in the decision-making and action-taking process. Voice has been defined as “expressing relevant ideas, information, and opinions about possible improvement” (12). Having the opportunity to share one’s voice may be valued by providers who practice with autonomy. Thus, because deliberative processes can be structured to accommodate the needs of specific clinics, engage the particular wisdom of those who would be affected by a decision, and enable providers with autonomy to share their voice, they may be effective approaches to identifying implementation interventions in settings characterized by clinic autonomy, provider autonomy, and individuals with diverse roles.

An evidence-based clinical practice guideline that is poorly adopted by providers is the American Dental Association’s (ADA) evidence-based pit-and-fissure dental sealant guideline (13). This guideline addresses using sealants for two purposes: to prevent tooth decay and to arrest tooth decay before it becomes cavitated (i.e., a cavity). Sealants are non-invasive and can have a big impact, because by preventing invasive treatments such as fillings, they preserve the integrity and increase the lifespan of the tooth. Despite the evidence-based support for dental sealants (14), most general dentists are not implementing the guideline (13). Adherence among general dentists to placing sealants on sound surfaces for prevention ranges from less than 5–38.5% (15, 16), and it is thought that adherence to placing sealants to arrest non-cavitated carious lesions (NCCLs) is even lower.

At its most basic, implementation involves identifying barriers, identifying an implementation strategy comprising multiple implementation interventions, and deploying that strategy (17). Based on our formative research in this organization, we identified two barriers to dentists placing sealants to arrest decay. First, dentists remained skeptical that sealants can arrest decay and were concerned that decay could progress under the sealant. Thus, they may have been experiencing a conflict between the guideline and their autonomy. Second, we found that the organization lacked a robust implementation process and relied on passive dissemination of the guideline. Despite tailoring the guideline to their clinical setting and revising their version of the guideline twice over the course of 15 years, dentists in the organization under study had not improved their adherence to the therapeutic portion of the guideline. Previous attempts to address the lack of adherence to the dental sealant guideline, such as by offering continuing education courses, had been top down and not clinic-specific. Thus, we believed that a deliberative engagement process could help clinic leadership identify more effective implementation interventions.

The purpose of this study was to determine whether by using a deliberative engagement process clinic leaders in an organization could identify an implementation strategy and providers could improve their adherence to a guideline based on the ADA’s pit-and-fissure dental sealant guideline. We hypothesized that by engaging in a deliberative engagement process, providers and staff would be able to form and share with leadership their opinions about implementation interventions. And we hypothesized that by learning providers’ and staffs’ opinions about implementation interventions, leaders would adopt a well-informed implementation strategy and providers would subsequently place sealants on more eligible lesions compared with prior to their participation. Furthermore, we hypothesized that forming an informed opinion and sharing one’s voice would mediate the relationship between experiencing the deliberative engagement process and placing sealants.

## Methods

### Study Design

The DISGO study used a cluster-randomized, stepped-wedge design. Based on both clinic volume and provider staffing full-time equivalents in 2019, we categorized clinics into tertiles according to size as large, medium, or small. One of the study analysts used the rand function in SAS on a Uniform(0, 1) distribution to order clinics within each tertile, numbering them 1 - 5. Clinics were assigned to five clusters defined by this ordering, with every cluster containing a single clinic from each tertile. One of the study project managers then enrolled clinics by the cluster number. We deployed the deliberative engagement process by cluster, allowing six weeks between each cluster. Prior to launching the deliberative engagement process in the clusters, we deployed it in one vanguard clinic. Each clinic had its own nonintervention and intervention periods. For the study as a whole, the nonintervention period began January 1, 2021, and the intervention period ended December 31, 2021. For each clinic, their intervention period began when the providers and staff in that clinic received a summary report of their recommendations following their deliberative forum. The deliberative engagement process was deployed in the vanguard clinic in March 2021 and in the final clinic of the last cluster in October 2021.

### Study Site and Sample

The Kaiser Permanente Dental (KPD) program is part of the Kaiser Permanente Northwest (KPNW) integrated health care system and provides comprehensive, prepaid dental care services to over 250,000 dental plan members in Oregon and southwest Washington. Across all KPD plans, there are no age or frequency limitations for placing dental sealants. In addition, KPD is part of Health Share of Oregon (www.healthshareoregon.org), a large coordinated care organization that services the state’s Medicaid population in the tri-county Portland metropolitan area.

Staff are employed in two different organizations. Dental hygienists, expanded function dental assistants, and administrative staff are employed by KPNW and are mostly unionized. Dentists and denturists are employed by Permanente Dental Associates (PDA), which is employee-owned and which has a contract with KPNW to provide dental services. Kaiser Permanente engages with its workforces through a labor-management partnership. In the dental clinics, this partnership is manifested in a self-directed decision-making structure called a Unit-Based Team (UBT), which is coled by a manager, a staff member, and a dentist. The team includes all the members of the work group, which enables it to build on the diverse perspectives and expertise of the team members. The purpose of the UBT is to address issues affecting performance or the work environment, and thus these meetings may serve as a facilitator to implementation.

We included all clinics that were not dedicated urgent care clinics. The deliberative engagement process targeted general and pediatric dentists, dental hygienists, expanded function dental assistants, and administrative staff in the clinics. Specialists who would not be likely to place sealants, such as periodontists, endodontists, and prosthodontists, were excluded.

### Deliberative Engagement Process

To address the first component of a deliberative engagement process, access to expert information providing background information about an issue, during a UBT meeting, clinic providers and staff watched a 15-minute prerecorded video introduction to the study and to the deliberative engagement process. Subsequently, via email, they received a 27-page workbook providing background material, the two barriers, and possible implementation interventions to consider (see the clinical protocol (18) and Additional File 1 for a description of the workbook).

To address the second component of the deliberative engagement process, participating in a facilitated small group discussion, and third component, considering options, about a month after receiving the background information, clinic providers and staff participated in an online, chat- (text-) based, facilitated small group discussion (i.e., deliberative forum) using the Common Ground for Action platform (Kettering Foundation and the National Issues Forums Institute, Dayton, Ohio). The target group size was 6-8, and to ensure role diversity within each group, clinic providers and staff were assigned to specific groups based on their role. Each discussion lasted 1 ½ hours, in place of a standing UBT meeting.

To address the fourth component of the deliberative engagement process, sharing informed opinions with leadership, immediately following each small group discussion, we provided clinic providers and staff a link to a Qualtrics survey (Qualtrics, Provo, Utah) where they could both record their recommendations to leadership and complete a survey about the process and their perception of voice. Although in the deliberative forum, eight possible implementation interventions were presented, on the post-session survey, we included only the five with the strongest evidence base as determined in our formative research (19). We summarized survey responses by clinic and provided 4-page reports to clinic leadership, clinic providers and staff, and practice leadership including the PDA Dental Director for Evidence-Based Practice.

### Evidence-Based Practice in the Guideline

The PDA guideline recommends that dentists and dental hygienists apply either of two sealant materials, resin or glass ionomer. If a resin sealant is applied, first the tooth surface is cleaned. Then the tooth is isolated, dried, and rinsed. After rinsing, the tooth is re-isolated and dried again. Then, the etching is checked, and the sealant is applied, cured with a light, and checked. If a glass ionomer sealant is applied, first the tooth is isolated and cleaned. Then, the sealant is applied and checked.

### Primary Outcome

The primary outcome, a measure of Reach, the extent to which a program attracts its intended audience (20), was calculated for each clinic and for each dentist as the difference in the rates of sealant application for occlusal, enamel carious lesions between the intervention and nonintervention periods. This represents patient-level receipt of guideline-concordant care. Providers’ rates of sealant application for occlusal enamel lesions were extracted from the electronic health record.

### Proximal Outcomes

These outcomes included survey respondents’ agreement with the two barriers we had identified and the implementation interventions we provided (see Additional File 2). These questions were scored on a 5-point Likert-type scale ranging from “Strongly Disagree” (coded 1) to “Strongly Agree” (coded 5) and were followed by free text response boxes for comments and suggestions. Clinic providers and staff answered these questions immediately following the deliberative forum, via the post-session survey.

### Mediators

We conducted exploratory analyses of three putative mediators: change in opinion, perception of voice, and adoption of clinic report recommendations. To measure change in each forum participant’s opinions, for each of the eight possible implementation interventions, we compared each forum participant’s ratings (i.e., ActionWeShould, ActionConflicted, ActionWeShouldNot, ActionUnranked) at the beginning of the deliberative forum with their ratings at the end of the deliberative forum.

We employed four measures of clinic providers’ and staffs’ perceptions of voice, which we define as having the opportunity to share one’s views. First and second, we administered the Promotive and Prohibitive Voice scales [21]. Clinic providers and staff completed the scales immediately following the deliberative forum, via the post-session survey. Clinic providers and staff completed the scales twice, first as the scales characterized their deliberative forum and second as the scales characterized past UBT meetings. Each scale has five items and is scored on a 5-point Likert-type scale ranging from “Strongly Disagree” (coded 1) to “Strongly Agree” (coded 5).

Third, the post-session survey included a question addressing the clinic providers’ and staffs’ perception of leadership responsiveness to feedback in the past year and anticipated responsiveness to the feedback from the forum. These questions were scored on a 5-point Likert-type scale ranging from “Strongly Disagree” (coded 1) to “Strongly Agree” (coded 5) and were followed by a free response box for comments and suggestions.

Fourth, we analyzed the transcripts from the deliberative forums using six codes to assess forum participant voice (results published separately; Grub, under review). An example of one of the codes is as follows: “Suggest new behaviors which are beneficial to my clinic.”

To understand the process of adoption of clinic report recommendations, we interviewed a key leader at each clinic, such as the clinic manager, three months after delivering the clinic report. To guide the interview we developed a set of 15 open-ended questions that included: “Tell me about your experience with the Deliberative [Engagement Process];” “Do you think the forum led to improved strategies and increased implementation of guidelines?;” and “Were there any conversations that took place after the forum, and can you tell me what was said?” For a description of the data analysis, please see the Additional File 3. One separate interview was conducted with the Dental Director for Evidence-Based Practice. This interview included six questions asking his thoughts about the summary reports provided to the clinics and any actions taken or planned as a result of those reports.

### Power

Power was calculated using the “stepped wedge” package in Stata 16 [15], based on the approach developed by Hussey and Hughes [16]. Our null hypothesis was that providers’ rates of placing or treatment planning sealants for occlusal NCCLs would not differ between the nonintervention and intervention periods. Our alternative hypothesis was that providers’ rates of placing or treatment planning sealants for occlusal NCCLs would increase following the intervention. We assumed a Type I error of 0.05, a 3% rate of placing or treatment planning sealants in the non-intervention period, and a 10% rate in the intervention period, which is an increase seen in similar interventions [17]. Randomizing 15 clinics along with 1 vanguard clinic using a stepped-wedge approach with these assumptions, we had at least 80% power to detect a difference and close to 100% power under some scenarios.

### Statistical Analysis

The primary end point was calculated as the change in the providers’ rates of placing or treatment planning sealants for occlusal NCCLs from before to after clinic providers and staff were exposed to the deliberative engagement intervention. The PDA guideline includes children, adolescents, and adults. To enable comparison with studies following the ADA guideline, in addition to analyzing lesions occurring in children, adolescents, and adults, we also conducted analyses for lesions occurring in just children and adolescents. Per the ADA, we defined “children and adolescents” as persons ranging in age from 6 through 17. The rates, and their difference, were treated as continuous variables. We used a generalized linear mixed model to model the intervention effect on sealant placement while nesting teeth within provider, and provider within clinic. We also accounted for secular time trends as a categorical fixed effect.

To examine differences between clinics and between survey respondents within clinics on agreement with the barriers and implementation interventions, we conducted ANOVA for each question to compare the mean scores by clinic (F-test) and the variance within clinic (Bartlett’s test). To quantify change in opinion from the beginning to the end of the deliberative forum, we calculated a Cohen’s kappa for each forum participant. Ratings were treated as changed if the rating made before the forum differed from the rating made at the end of the forum. We used descriptive statistics to summarize the distribution of the Cohen’s kappas and the voice self-reports. All analyses were conducted using Stata 17 (College Station, TX) and RStudio.

### SARS-CoV-19 Modifications

Prior to the start of the intervention, due to SARS-CoV-19 pandemic restrictions on in-person meetings, we changed the setting of the deliberative forums from an in-person meeting to the Common Ground for Action online platform. Because the clinics were closed for several months at the beginning of the pandemic, we started the intervention 6 months later than we had originally intended, and as a result, we reduced the follow-up time from 12 months to 6 months. To accommodate the reduction in follow-up time, we changed the design of the study from a randomized, controlled clinical trial to a cluster-randomized, stepped wedge trial. Additionally, we expanded the primary outcome from sealants placed to sealants placed or treatment planned.

## Results

### Proportion Recruited and Characteristics of the Providers Eligible to Participate in the Deliberative Engagement Process

During January – March 2021, over 65% of the staff were White, and 99% were non-Hispanic (see Table 1). For the KP Dental staff, the numbers reflect all staff at dental offices except for four combined dental/medical offices. For the combined offices, we included staff who report through Dental Operations. Both PDA and KPD numbers are systemwide and include staff in non-participating clinics. For a given clinic, about one third of its staff are scheduled to be present on a given day. Across all clinics, about 44.3% of the entire staff, not just those scheduled, participated in an introductory session. About 40.5% of the entire staff participated in a deliberative forum. And about 28.2% of the entire staff completed a post-session survey (see Table 2). Over the course of 2021, 15 dentists were hired by PDA, and seven left PDA.

### Characteristics of the Patients Eligible to Receive Sealants for NCCLs

There were 7 clinics with a pediatric practice and 9 that were adult-only. Over 48% of the patients with occlusal NCCLs were ages 31-64 (see Table 3). Over 55% were female; close to 80% were non-Hispanic; and over 60% were White.

### Primary Outcome of the Deliberative Engagement Process: Rate of Sealants Placed on Occlusal NCCLs

During the non-intervention period, sealants were placed on 2.3% of the eligible lesions (primary: 1.9%; permanent: 2.3%; see Table 4). During the intervention period, sealants were placed on 2.6% of the eligible lesions (primary: 1.5%; permanent: 2.9%). We found the OR = 0.80, with 95% CI (0.58-1.11), p=0.18 (see Table 4). Thus, no statistically significant change was observed from the non-intervention period to the intervention period. Similar results were obtained when examining children only (see Table 4). Because the OR was less than 1 even though the raw percentage had increased, we conducted post hoc analyses. In post hoc analyses, we found that one clinic had an outsized impact. That clinic was responsible for about 1/3 of the sealants placed during the study and just over 50% of the sealants placed in the intervention period. However, their sealant placement rate went down from 9.9% in the non-intervention period to 7.9% in the intervention period. Overall, we found that 6 clinics had a higher rate in the intervention period, 8 had a lower rate, and 2 were unchanged.

### Proximal Outcomes: Clinic Endorsement of Barriers and Implementation Interventions

**Figure 1:**
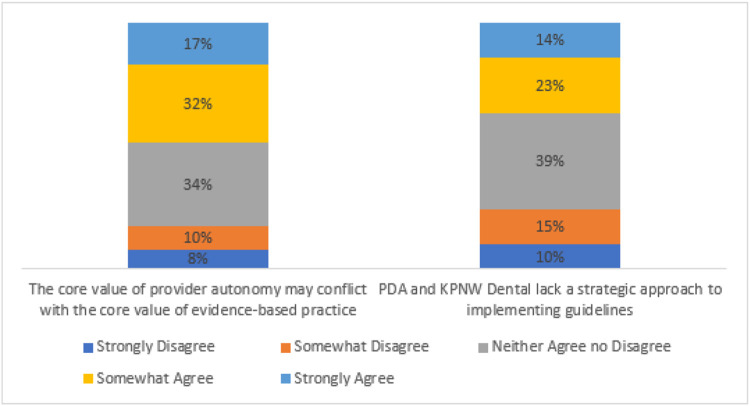
Strength of survey respondents’ endorsement of barriers

**Figure 2:**
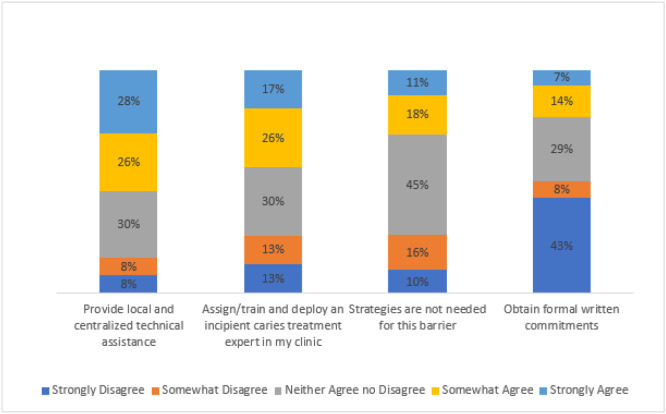
Strength of survey respondents’ endorsement of implementation interventions for the barrier: “The core value of provider autonomy may conflict with the core value of evidence-based practice”

**Figure 3:**
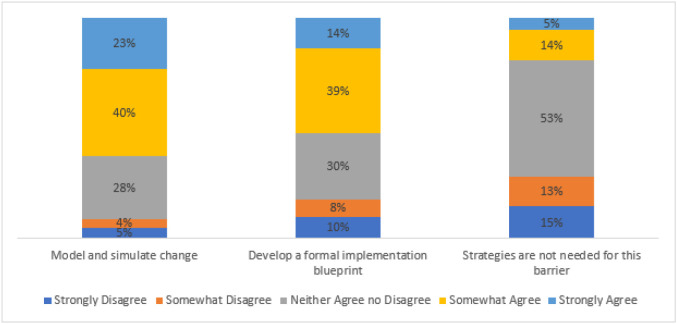
Strength of survey respondents’ endorsement of implementation interventions for the barrier: “PDA and KPNW Dental lack a strategic approach to implementing guidelines”

Across the clinics, 49% of survey respondents strongly or somewhat strongly agreed on the post-session survey that the core value of provider autonomy may conflict with the core value of evidence-based practice (see Figure 1). Among survey respondents who endorsed this barrier, the implementation intervention they most frequently endorsed as strongly or somewhat agree (54%) was “provide local and centralized technical assistance” (see Figure 2). With respect to the second barrier, across the clinics, 37% of survey respondents strongly or somewhat strongly agreed that PDA and KPNW Dental lack a strategic approach to implementing guidelines (see Figure 1). Among survey respondents who endorsed this barrier, the implementation intervention they most frequently endorsed as strongly or somewhat agree (63%) was “model and simulate change” (see Figure 3). Forty-three survey respondents wrote in other barriers.

There was no difference between clinics or within clinics on the mean ratings of the barriers. With respect to the implementation interventions, clinics differed in the degree to which they endorsed all three of the implementation interventions targeting barrier 1, “The core value of provider autonomy may conflict with the core value of evidence-based practice” (see Table 5). Compared with small clinics, medium and large clinics endorsed greater agreement with the implementation interventions, “Assign/train and deploy an incipient caries treatment expert in my clinic,” F(2,193) = 3.29, p < .04, and “Obtain formal written commitments,” F(2,191) = 4.99, p < .01. Clinics also differed regarding the implementation intervention, “Provide local and centralized technical assistance,” F(15,178) = 2.07, p < .01, although this difference was not associated with the size of the clinic. The within clinic variability was not significantly different for any of the implementation interventions. Thirteen survey respondents wrote in other implementation interventions for this barrier.

Clinics differed in the degree to which they endorsed one of the two implementation interventions targeting barrier 2, “PDA and KPNW Dental lack a strategic approach to implementing guidelines.” Compared with small clinics, medium and large clinics endorsed greater agreement with the implementation interventions, “Develop a formal implementation blueprint,” F(2,175) = 4.59, p < .01. The within clinic variability was not significantly different for either of the implementation interventions. Eight survey respondents wrote in other implementation interventions for this barrier.

### Mechanisms of Action

#### Informed Opinion

**Figure 4:**
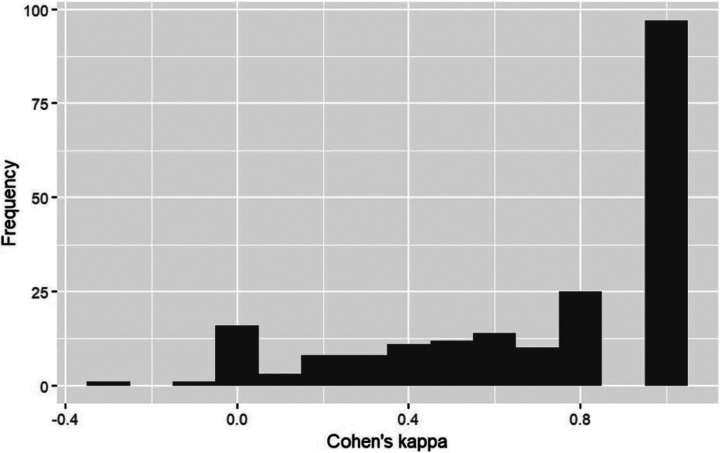
Change in forum participants’ endorsement of possible implementation interventions from the beginning to the end of the deliberative forum as measured by person-level Cohen’s kappas.

We found little change in forum participants’ ratings of the eight implementation interventions from the beginning to the end of the deliberative forum. The mean Cohen’s kappa was 0.80; the median was 0.71; and the range was −0.27 – 1.00. The distribution was skewed to the left (see Figure 4).

#### Voice

Survey respondents neither agreed nor disagreed they shared both promotive and prohibitive voice during the deliberative forum and somewhat agreed during previous UBT meetings. For promotive voice, we obtained the following values: M_(deliberative forum)_ = 3.31, SD_(deliberative forum)_ = 1.08, and M_(UBT)_ = 4.09, SD_(UBT)_ = 0.86. For prohibitive voice, we obtained the following values: M_(deliberative forum)_ = 3.14, SD_(deliberative forum)_ = 1.00, and M_(UBT)_ = 3.65, SD_(UBT)_ = 0.89. Survey respondents somewhat agreed that leadership would take their suggestions into consideration: M(_deliberative forum)_ = 3.43, SD_(deliberative forum)_ = 1.13, and M_(UBT)_ = 3.69, SD_(UBT)_ = 1.00.

#### Adoption of Clinic Report Recommendations

For the three-month follow-up interviews, at 12 clinics, 1 leader was interviewed; at two clinics, 2 leaders were interviewed; and for two clinics, 1 leader provided one interview. The sample consisted of 14 females and 3 males (3 dentists and 14 administrative staff members). One separate interview was conducted with the Dental Director for Evidence-Based Practice.

Actions leaders described as having occurred included sharing the clinic’s summary report with staff, discussing the post-session survey results in UBT meetings, and having informal discussions about placing sealants. In their interviews, leaders did not mention taking steps to implement any of the implementation interventions discussed in the deliberative forums. Despite interview questions focusing on actions taken following the deliberative forums, the leaders primarily provided information about barriers faced and feedback on the deliberative engagement process. Three main barriers to taking action were identified, including challenges in diagnosing eligible lesions, concern about staffs’ skills in placing sealants, and workflow issues stemming from time and staffing shortages. Some leaders misperceived that the procedure being targeted for implementation was placing sealants to prevent decay not to arrest decay.

The Dental Director for Evidence-Based Practice reported that he read all the summary reports and followed up with clinic leaders about the reports. Actions he planned but had not yet taken included training the dentists in how to diagnose NCCLs and providing more information about the evidence supporting placing sealants to arrest the decay process. He reported hearing from clinic leaders about barriers to placing sealants including lack of clarity about how to diagnose an NCCL, the importance of provider autonomy in determining the treatment plan, and lack of acceptance of the evidence supporting placing sealants for NCCLs. He also said that the culture around quality improvement in the clinics was to move on from one issue to another without sustained attention once training had been completed.

### Fidelity to the Deliberative Engagement Process as Planned, Adaptations, and Important Harms or Unintended Effects

We administered 100% of the introductory sessions and deliberative forums and distributed the workbooks to all clinic staff. We administered post-session surveys after each deliberative forum. We provided all clinics and leadership with their reports summarizing survey results. We did not have to make any adaptations to the deliberative engagement process during the intervention period. No known important harms or unintended effects occurred during the study.

## Discussion

The purpose of this study was to determine whether a large group dental practice with semi-autonomous clinics, providers with autonomy, and staff representing many professional roles could use a deliberative engagement process to endorse clinic-specific implementation interventions and increase providers’ adherence to the PDA’s pit-and-fissure guideline. We found that the size of the clinic was associated with how strongly respondents agreed that their clinic should adopt two of the three proposed implementation interventions targeting the first barrier and one of the two proposed implementation interventions targeting the second barrier. Following the deliberative engagement process, however, providers did not place more sealants on occlusal NCCLs compared with the non-intervention period.

We also examined mechanisms by which the deliberative engagement process may have influenced respondents’ implementation intervention endorsement. These included perceptions of voice, the presence of a range of opinions, and change in opinion. We found that survey respondents endorsed being able to share both promotive and prohibitive voice during the forums. However, we found no evidence forum participants developed a range of opinions and little change in their opinions about the implementation interventions from the beginning to the end of the forum.

### Rate of Placing Sealants

The deliberative engagement process did not change dentists’ rate of placing sealants on occlusal NCCLs. There are many intervening factors between the end of the deliberative engagement process and dentists' treatment planning or placing a sealant on a patient’s occlusal NCCL. Issues in these intervening factors may have contributed to the lack of change in sealant placement rate. For example, in our three-month follow-up interviews, we learned that clinic leadership did not adopt any implementation interventions. Future research should identify issues in these intervening factors and possible solutions. It is likely that any successful implementation strategy will take the form of a bundle of implementation interventions each addressing a step in the process of implementation.

### Recommendations for Implementation Interventions to Adopt

The deliberative engagement process had a proximal effect on the survey respondents’ recommendations for implementation interventions to adopt in their clinics. Compared with small clinics, medium and large clinics more strongly agreed that their clinic should adopt two of the three proposed implementation interventions targeting the first barrier and one of the two proposed implementation interventions targeting the second barrier. Specifically, with respect to the barrier, “the core value of provider autonomy may conflict with the core value of evidence-based practice,” survey respondents in the medium and large clinics more strongly agreed that their clinic should assign/train and deploy an incipient caries treatment expert. Although the survey respondents in the medium and large clinics also viewed the implementation intervention “obtain formal written commitments” more favorably than small clinics, none of the clinics endorsed this implementation intervention. With respect to the second barrier, “PDA and KPNW Dental lack a strategic approach to implementing guidelines,” survey respondents in the medium and large clinics more strongly agreed that their clinic should develop a formal implementation blueprint. Thus, the staff’s support for implementation interventions may vary depending on the size of the clinic. The sources of this similarity and dissimilarity across clinics should be identified in future research.

### Mechanisms of Action: Voice, Diverse Opinions, Change in Opinion

With respect to the effects of the deliberative engagement intervention on the mechanisms it targets, we found mixed results. Providers accustomed to working with autonomy may value an intervention that enables them to express their views. In both our qualitative analysis of the forum chats (Gruß, under review) and quantitative analysis of the post-session self-report questionnaires reported here, we found evidence that forum participants and survey respondents shared both promotive and prohibitive voice. This means that they were expressing ways to improve their clinics and also concerns about practices that could be harmful to their clinics. Thus, forum participants and survey respondents achieved this necessary condition to engage difference as a resource.

Second, we expected that forum participants would have diverse views to share, by virtue of their different professional roles. However, we did not find evidence that at the end of the forums, the forum participants had different opinions about the implementation interventions. There was no significant within-group variability. It is unclear whether forum participants shared similar views or whether by the end of the forums, they achieved consensus. Future research should examine whether colleagues who work together share similar views or whether diverse views were shared.

Third, we hypothesized that the process of hearing diverse views would lead forum participants to develop a more informed opinion about the implementation intervention from the beginning to the end of the forum. However, the majority of forum participants did not change their opinions over this time. It is possible that participants changed their opinions following exposure to the introductory session and workbook, to which our measure was not sensitive. Alternatively, participants could have become more informed without changing their opinions. Finally, if the views that participants shared during the forums were not diverse, then their opinions would not change. Thus, we were not able to evaluate the hypothesis completely.

### Strengths

One strength of the study was that we obtained many process measures, which enabled us to evaluate whether deliberative engagement affected the mechanisms by which it is proposed to work. Additionally, we designed the deliberative engagement to target barriers and build on facilitators identified according to the COM-B model of behavior change. Finally, we were able to administer the intervention with complete fidelity.

### Limitations

Deliberation in UBT meetings involves in-person, verbal engagement, whereas, in our study, participants had to use an unfamiliar, chat-based tool (i.e., the CGA online platform). Additional practice with the online platform or holding the forums in person could overcome this limitation.

We conducted the intervention during the SARS-CoV-2 pandemic, and this may have affected our ability to evaluate the intervention fairly. During the time of our study, the clinics experienced staffing shortages and low morale as a consequence of the SARS-CoV-2 pandemic. This could have adversely affected our ability to evaluate the deliberative engagement process. On the other hand, there are several reasons why it might have been a fair evaluation. The barriers preventing guideline adoption predated and were not affected by the pandemic. In response to the pandemic, dentists initially were not allowed to place sealants, but this restriction had ended by the time the intervention began. Additionally, in response to the pandemic, clinic UBT meetings shifted from being in person to being virtual. Thus, it is unclear whether the pandemic affected our evaluation of the deliberative engagement intervention.

### Future Directions

First, it will be important to determine whether the lack of a range of perspectives among forum participants is a feature of colleagues who work together or whether it occurs under some conditions but not others. Second, throughout the study, providers and staff articulated a number of barriers. More work may need to be done to identify and obtain consensus regarding the important barriers. We would be interested to determine whether a deliberative process could assist with this step. Finally, leadership may need assistance in deploying selected implementation interventions.

## Conclusion

In sum, we conclude that a deliberative engagement intervention may help clinic leadership identify implementation interventions when there is a challenging problem, a network of semi-autonomous clinics, and autonomous providers within those clinics. It remains to be determined whether there is a range of perspectives among clinic staff. For clinics that have a challenging problem and a culture of autonomy however, a deliberative engagement process warrants future study as one component in developing an implementation strategy. The deliberative approach is a novel way to obtain clinic provider and staff perspectives, an aspect of implementation efforts that has been identified as being important (21) but for which there currently exist few options, and to identify implementation interventions. Employing it in a clinical setting extends the deliberative approach into a new realm.

## Figures and Tables

**Table 1.** Characteristics of the recipient population for the deliberative engagement process, the KPNW dental office staff: dentists, dental hygienists, expanded function dental assistants, and administrative staff

Characteristic	Dentists N (%)	Dental Hygienists, Expanded Function Dental Assistants, and Administrative Staff N (%)
All participants	172 (100)	508 (100)
Age (y)		
18 - 30	2 (1)	64 (13)
31 - 64	164 (95)	441 (86)
65+	6 (4)	3 (1)
Overall average age and range	45 (28 – 71)	44 (20 – 70)
Gender		
Male	96 (56)	35 (7)
Female	76 (44)	473 (93)
Non-binary (if known)	0 (0)	0 (0)
Ethnicity		
Hispanic or Latino	2 (1)	43 (8)
Not Hispanic or Latino	170 (99)	465 (92)
Unknown/unreported	0 (0)	0 (0)
Race		
American Indian or Alaska Native	1 (1)	12 (2)
Asian	45 (26)	52 (10)
Black or African American	2 (1)	24 (5)
Native Hawaiian or Other Pacific Islander	1 (1)	5 (1)
Two or more races	8 (5)	0 (0)
White	115 (67)	405 (80)
Unknown/unreported	0 (0)	10 (2)

*Note.* Sources: Permanente Dental Associates and Kaiser Permanente Dental Plan administrative records for Jan – Mar 2021. Percentages may not equal totals due to rounding.

**Table 2.** Number of Permanente Dental Associates dentists and Kaiser Permanente Dental administrative staff, expanded function dental assistants, dental hygienists recruited

Clinic	Size	Clinic Census	Intro Session Attendance	# Forums Scheduled & Held	Forum Planned Attendance	Forum Actual Attendance	# Surveys Received	Survey Response Rate
Vanguard	Vanguard	47	48	4	25	25	8	17%
Cluster 1								
1	L	91	46	7	45	46	33	36%
2	S	31	14	3	17	16	18	58%
3	M	44	20	3	21	27	19	43%
Cluster 2								
4	S	45	22	3	19	18	13	29%
5	M	43	22	4	24	22	13	30%
6	L	102	39	6	38-40	36	29	28%
Cluster 3								
7	L	60	33	5	33	37	24	40%
8	M	89	27	3	22	20	12	13%
9	S	15	8	1	8	7	6	40%
Cluster 4								
11	L	67	22	4	22	22	20	30%
12	M	33	20	4	25	17	15	45%
14	S	31	18	3	15	15	13	42%
Cluster 5								
13	L	66	20	4	26	20	8	12%
10	S	90	16	3	16	15	7	8%
15	M	42	22	4	21	20	15	36%
TOTAL		896	397	61	377-379	363	253	28%

Note. At the start of the study, the overall16 intervention clinic census totaled 896 dental providers and staff. At the end of the study, we received demographics of dental providers and staff at the same clinics and received data for 680. We were informed that some staff members are considered "floaters." Floaters are not assigned to one clinic, but instead work at several and rotate depending on need. Floaters may have been counted several times, resulting in this census discrepancy.

**Table 3.** Characteristics of patients with enamel lesions on occlusal surfaces of molar teeth

	N (Percentage)		
Patient Characteristic	Overall (n=5334)	Primary (n=612)	Permanent (n=4,722)
Age (y)			
0 – 5	0 (0)	0 (0)	0 (0.0)
6 - 12	791 (14.8)	549 (89.7)	242 (5.1)
13 - 17	597 (11.2)	54 (8.8)	543 (11.5)
18 - 30	1,127 (21.1)	2 (0.3)	1,125 (23.8)
31 - 64	2,585 (48.5)	5 (0.8)	2,580 (54.6)
65+	234 (4.4)	2 (0.3)	232 (4.9)
Gender			
Male	2,264 (42.4)	299 (48.9)	1,965 (41.6)
Female	3,070 (57.6)	313 (51.1)	2,757 (58.4)
Non-binary	0 (0.0)	0 (0.0)	0 (0.0)
Ethnicity			
Hispanic or Latino	545 (10.2)	70 (11.4)	475 (10.1)
Not Hispanic or Latino	4,264 (79.9)	473 (77.3)	3,791 (80.3)
Unknown	525 (9.8)	69 (11.3)	456 (9.7)
Race			
American Indian or Alaska Native	20 (0.4)	4 (0.7)	16 (0.3)
Asian	474 (8.9)	61 (10.0)	413 (8.8)
Black or African American	225 (4.2)	23 (3.8)	202 (4.3)
Native Hawaiian or Other Pacific Islander	40 (0.8)	0 (0.0)	40 (0.9)
White	3,444 (64.6)	373 (61.0)	3,071 (65.0)
More than one race	143 (2.7)	18 (2.9)	125 (2.7)
Unknown	943 (17.7)	129 (21.1)	814 (17.2)
Other	45 (0.8)	4 (0.7)	41 (0.9)

*Note*. This represents the number of unique patients and not the number of tooth surfaces analyzed.

**Table 4.** Number and percentage of occlusal enamel (i.e., non-cavitated) carious lesions (NCCLs) receiving a sealant before and after the deliberative engagement process

Sealant Placed	Non-Intervention Period	Intervention Period	Total
	Total		
No	5,846 (97.7%)	3,522 (97.4%)	9,368 (97.6%)
Yes	137 (2.3%)	95 (2.6%)	232 (2.4%)
Total	5,983 (100.0%)	3,617 (100.0%)	9,600 (100.0%)
	Primary Teeth		
No	666 (98.1%)	766 (98.5%)	1,432 (98.3%)
Yes	13 (1.9%)	12 (1.5%)	25 (1.7%)
Total	679 (100.0%)	778 (100.0%)	1,457 (100.0%)
	Permanent teeth		
No	5,180 (97.7%)	2,756 (97.1%)	7,936 (97.5%)
Yes	124 (2.3%)	83 (2.9%)	207 (2.5%)
Total	5,304 (100.0%)	2,839 (100.0%)	8,143 (100.0%)
	Children Ages 6 - 17		
No	1,459 (92.4%)	1,266 (93.9%)	2,725 (93.1%)
Yes	120 (7.6%)	82 (6.1%)	202 (6.9%)
Total	1,579 (100.0%)	1,348 (100.0%)	2,927 (100.0%)

**Table 5.** Means, Standard Deviations, and One-Way Analyses of Variance of Agreement with Implementation Interventions by Clinic Size

Implementation Strategy	Small		Medium		Large		*F*	df	h^2^
	*M*	*SD*	*M*	*SD*	*M*	*SD*			
	Barrier 1: The core value of provider autonomy may conflict with the core value of evidence-based practice								
Assign/train and deploy an incipient caries treatment expert in my clinic	1.80	1.17	2.35	1.22	2.33	1.29	3.29*	2,193	0.03
Provide local and centralized technical assistance	2.50	1.11	2.72	1.09	2.51	1.34	0.65	2,191	0.01
Obtain formal written commitments	0.91	1.19	1.70	1.34	1.30	1.33	4.99**	2,191	0.50
Strategies are not needed for this barrier	2.06	1.10	2.13	1.12	1.92	1.09	0.54	2,149	0.01
	Barrier 2: PDA and KPNW Dental lack a strategic approach to implementing guidelines								
Develop a formal implementation blueprint	2.05	1.28	2.73	1.03	2.31	1.08	4.59*	2,175	0.50
Model and simulate change	2.72	1.10	2.85	0.86	2.65	1.09	0.63	2,175	0.01
Strategies are not needed for this barrier	1.79	0.99	1.88	0.98	1.80	1.09	0.09	2,126	0.00

Note:

* *p*<.05

** *p*< .01.

0 = “Strongly Disagree,” 1 = “Somewhat Disagree,” 2 = “Neither Agree Nor Disagree,” 3 = Somewhat Agree,” 4 = “Strongly Agree.”

## Abbreviations

ADA: American Dental Association
CGA: Common Ground for Action
COM-B: Capability, Opportunity, Motivation - Behavior
KPD: Kaiser Permanente Dental
KPNW: Kaiser Permanente Northwest
NCCL: Non-cavitated carious lesion
PDA: Permanente Dental Associates
UBT: Unit based team
